# Multivariate time series dataset for space weather data analytics

**DOI:** 10.1038/s41597-020-0548-x

**Published:** 2020-07-10

**Authors:** Rafal A. Angryk, Petrus C. Martens, Berkay Aydin, Dustin Kempton, Sushant S. Mahajan, Sunitha Basodi, Azim Ahmadzadeh, Xumin Cai, Soukaina Filali Boubrahimi, Shah Muhammad Hamdi, Michael A. Schuh, Manolis K. Georgoulis

**Affiliations:** 1 Department of Computer Science, Georgia State University, Atlanta, United States; 2 Department of Physics & Astronomy, Georgia State University, Atlanta, United States; 3 RCAAM of the Academy of Athens, Athens, Greece

**Keywords:** Space physics, Solar physics

## Abstract

We introduce and make openly accessible a comprehensive, multivariate time series (MVTS) dataset extracted from solar photospheric vector magnetograms in Spaceweather HMI Active Region Patch (SHARP) series. Our dataset also includes a cross-checked NOAA solar flare catalog that immediately facilitates solar flare prediction efforts. We discuss methods used for data collection, cleaning and pre-processing of the solar active region and flare data, and we further describe a novel data integration and sampling methodology. Our dataset covers 4,098 MVTS data collections from active regions occurring between May 2010 and December 2018, includes 51 flare-predictive parameters, and integrates over 10,000 flare reports. Potential directions toward expansion of the time series, either “horizontally” – by adding more prediction-specific parameters, or “vertically” – by generalizing flare into integrated solar eruption prediction, are also explained. The immediate tasks enabled by the disseminated dataset include: optimization of solar flare prediction and detailed investigation for elusive flare predictors or precursors, with both operational (research-to-operations), and basic research (operations-to-research) benefits potentially following in the future.

## Abstract (summary)

**Table:** 

Measurement(s)	solar flare • stellar radiation • solar magnetic data
Technology Type(s)	digital curation
Factor Type(s)	temporal interval • flare class • location
Sample Characteristic - Environment	star • climate system

Machine-accessible metadata file describing the reported data: 10.6084/m9.figshare.12444884

## Background & Summary

Solar flares and coronal mass ejections (CMEs)^1–3^ are events occurring in the solar corona and heliosphere that can have a major negative impact on our technology-dependent society^4^. A flare is characterized by a sudden brightening by orders of magnitude in Extreme Ultra-Violet (EUV) and X-ray and, for large events, gamma-ray emissions, from a small area on the Sun, lasting from minutes to a few hours. High-frequency electromagnetic radiation and particles from solar flares and eruptions are filtered out by Earth’s atmosphere, but they pose a hazard to astronauts and sensitive equipment in space. A strong enough CME can induce currents in the Earth’s atmosphere and large networks of conductive materials such as power grids, leading to surges, tripping, and melting of transformers.

A 2008 report by the National Research Council concluded that a solar superstorm similar to the 1859 Carrington event^5^ could cripple the entire US power grid for months and cause an economic damage of 1 to 2 trillion dollars^6^. In response, the White House released the National Space Weather Strategy and Space Weather Action Plan^4^ in 2015 as a roadmap for research aimed at predicting and mitigating the effects of solar eruptive activity. The plan suggests leveraging machine learning for space weather predictions, with vested interest in this recommended approach reiterated recently^7^. Key for this approach is to produce benchmark datasets for testing flare prediction algorithms, as mentioned in^8,9^.

The benchmark dataset described in this work is intended as a testbed for solar physicists or machine learning practitioners, by providing a cleaned, integrated, and readily available dataset with data verified from multiple sources. Successful flare predictions via machine learning models trained and tested on this dataset intend to (1) tackle a central problem in space weather forecasting and (2) help identify physical mechanisms pertaining, or even giving rise, to solar flares. This dataset is a reliable resource for providing an unbiased comparison between results from various solar flare prediction algorithms. Without the use of a fixed dataset, such as the one presented here, discrepancies in performance evaluation metrics between different machine learning methods cannot be attributed unambiguously to the differences in the dataset or the quality of the methods at hand.

Our benchmark dataset mainly relies on Spaceweather HMI Active Region Patches (SHARPs)^10^ available from the Joint Science Operations Center (JSOC). This product stems from solar vector magnetograms obtained by the Helioseismic Magnetic Imager (HMI)^11^ onboard the Solar Dynamics Observatory (SDO)^12^. HMI observes the Sun almost continuously and provides information on the magnetic field in the solar photosphere.

Since the cause of a solar flare is the sudden release of magnetic energy in the solar corona (see, e.g.^13–18^, - see also^1,19^ for comprehensive reviews) it makes sense to use available magnetic field information for modeling and flare prediction^20,21^. However, much of the HMI data is irrelevant for flare prediction since flares are known to originate from active regions; namely, areas of high concentration of magnetic flux. Thus, HMI active region patches were first created^22^. The HARP is a data pipeline product that identifies and tracks active regions in the solar photosphere, providing trimmed vector magnetic field maps. HARPs were then enriched with metadata (i.e., physical parameters inferred by magnetograms) of space weather forecasting interest, giving rise to Space Weather HARPs, or SHARPs^10^.

Information on possible flares occurring in the region of interest, however, is missing from the SHARPs. The National Oceanic and Atmospheric Administration (NOAA) operates Geostationary Operational Environmental Satellites (GOES) that have X-ray and particle detectors onboard. Since 1975, GOES have been detecting solar flares, and a catalog of all detected flares is available from NOAA^23^ while flare reports are available through the Heliophysics Events Knowledgebase (HEK)^24^. These flares are classified logarithmically via their peak X-ray flux as A, B, C, M and X. The GOES flare catalog contains the flare time (start, peak, end), GOES class, peak X-ray flux, a spatial location on the solar disk, and associated NOAA active region (AR) number, where available. Additionally, the Solar Region Summary (SRS) product provides daily data on NOAA-numbered ARs, including mean location and sunspot classification.

Flares have also been automatically detected by various solar feature detection modules^25^, and are regularly collected in various databases. These modules include Flare Detective^26^, SSW Latest Events^27^, RHESSI^28^ and Hinode flare observations^29^. Reports from two of these modules, SSW Latest Events and Hinode Flare Catalog, are used here as auxiliary data sources to verify the missing locations of flares in the GOES catalog. The methods utilized in the process of cleaning, verifying, and combining the individual flare source data are described in the following section.

## Methods

**Fig. 1:**
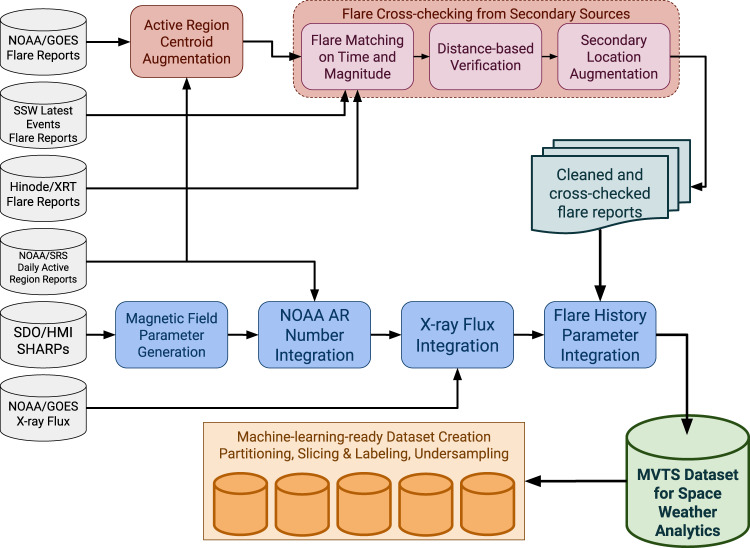
The block diagram of our dataset generation process, with principal procedures of flare cleaning (in red), MVTS generation and flare integration (in blue), and the eventual machine-learning-ready dataset creation (in orange).

Creating benchmark datasets for solar flare prediction based on magnetic maps of the Sun’s surface is a three-fold problem: first, solar flare reports from GOES need to be cleaned, with conflicting information resolved. Second, solar flare reports need to be matched with solar magnetic data. This can be done by either utilizing available NOAA AR numbers, if matched to HARP numbers present in SHARPs, or by performing a spatiotemporal overlap procedure between the onset time and location of a flare and the bounding box of an HMI active region patch (HARP) at that given time. Finally, sampling biases need to be eliminated when creating labeled datasets for training machine learning models. A schematic overview of the overall MVTS dataset generation process is presented in Fig. 1.

**Fig. 2:**
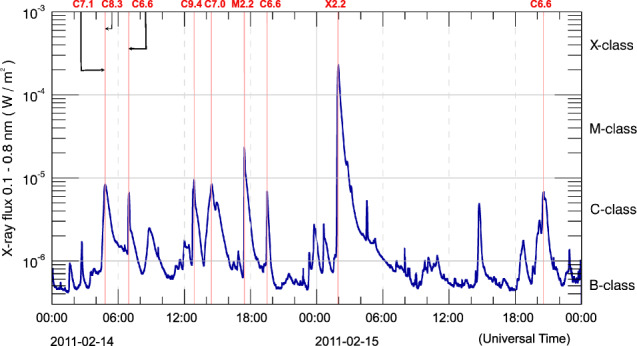
GOES15 1-8 Å solar X-ray flux from 2011-02-14 to 2011-02-15. The GOES flare classification is provided on the minor y-axis. The plot also includes annotations of flares exceeding GOES class C5.0, with red vertical lines indicating the flares’ peak time. The example interval also shows that during these two days of intense activity background X-ray flux was high, making it difficult to identify small flares. Notice also that the first two C-class flares peak essentially simultaneously (i.e., within 1 minute from each other).

**Fig. 3:**
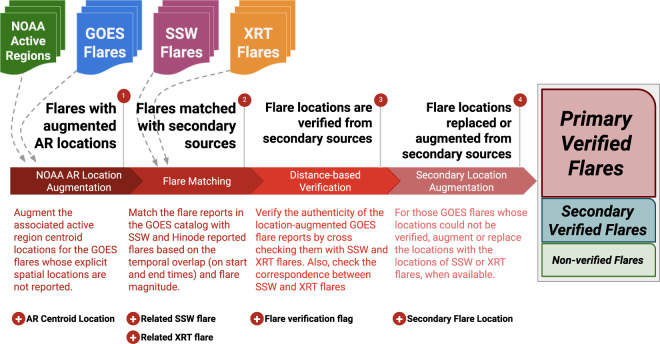
Overview of our 4-step flare data enhancement and cross-cheking procedures as well as accompanied enhancements after each step (brief explanations also provided). The cross-checking with secondary flare data sources (SSW Latest Events and Hinode-XRT) results in three sets of flare reports: (1) primary-verified, where the locations of the primary flare reports (from GOES) are verified by at least one secondary source; (2) secondary-verified, where GOES reported locations could not be verified but SSW and XRT reported locations are in agreement; and (3) non-verified, where flare location from any of the three data sources cannot be verified.

### Curating the NOAA active region database and NOAA-to-HARP associations

The list of NOAA active regions is a fundamental component of our data integration methodology. We use the NOAA active region (AR) locations to augment unknown or unreported flare locations (described in the next section). More importantly, the NOAA AR numbers are utilized for the integration of flare annotations to our MVTS through the HARP to NOAA AR number. Each HARPNUM (identifier of HARPs) is associated with zero, one, or more NOAA AR numbers. The list of HARPNUM associated to NOAA AR numbers is provided by JSOC and is available online in^30^. However, we have identified two issues with NOAA ARs: there are (1) a number of instances where NOAA AR daily reports have unexpected location changes and (2) instances of faulty associations, where either NOAA AR numbers were not associated with a HARPNUM when they should be, or vice versa.

As was described in^31^, to identify unexpected location reports, we utilize the daily heliographic latitude and longitude (the latter expressed as central meridian distance) changes for each NOAA AR report. Given this information, we identify abnormal location changes in the data by binning them based on their latitudes. The binning process utilized four groups of latitudinal zones covering the entire earthward solar hemisphere. These zones have absolute latitudes (i.e., repeated in solar North and South) (0°, 10°), (10°, 20°), (20°, 30°) and (30°, 90°). We found the median longitudinal displacement for each of these, and their distributions are shown in Fig. S.1 (in Supplementary File). The active regions were generally found to move between 13° to 14° westward daily, due to the solar differential rotation, as consistent with expectations. However, in cases identified as outliers we found that some active regions either did not change location or moved over 25° on a single day. Similarly, we observed outliers for latitudes, where the active region latitude changed over 5° on a single day.

Three example cases of anomalous NOAA AR movements and our corrections are shown in Fig. S.2 (in Supplementary File). Most of these outliers can be explained by a single misreporting, often in the first or the last observation close to the limbs, as shown in Fig. S.2(a,b). However, in some cases, the error propagated through the end of active region’s lifespan and multiple records had to be fixed; see the example in Fig. S.2(c). In Table S.1 (provided in Supplementary File), we show these identified NOAA AR daily report outliers and present their updated locations. In total, we have fixed the locations of 59 active regions.

Based on the updated NOAA AR locations, we then performed a spatiotemporal co-occurrence analysis between NOAA ARs and HARP locations, as described in^31^. The NOAA AR centroid locations are reported daily as a point coordinate. HARPs have bounding boxes reported every 12 minutes. We extrapolate the locations of the NOAA ARs based on the known solar differential rotation, using ±12 hours for every daily NOAA SRS report. Then, for each of these records, we check the temporal co-existence and spatiotemporal co-occurrence intervals between NOAA ARs and reportedly associated HARPs. Note that (temporal) co-existence refers to the time ranges where both NOAA ARs and HARPs are reported, while (spatiotemporal) co-occurrence refers to the times where a NOAA AR and a HARP co-exist, and the point coordinate of the NOAA AR lies within the HARP bounding box. Using this information, we calculate a co-occurrence factor (*cof*) defined as

1 $$cof=\frac{{\rm{Length}}\,{\rm{of}}\,{\rm{co}} \mbox{-} {\rm{existence}}\,{\rm{window}}}{{\rm{Length}}\,{\rm{of}}\,{\rm{co}} \mbox{-} {\rm{occurrence}}\,{\rm{window}}}.$$

In addition, we calculated the average minimum distance between the NOAA AR coordinate and the HARP bounding box during the time intervals they co-exist, which is denoted as *μ*_*mindist*_. For this calculation, the distance is calculated between the interpolated NOAA AR coordinate and the nearest point along the edge of the HARP bounding box, with NOAA AR coordinates either inside or touching the HARP bounding box considered to have a zero distance.

While calculating these values, we determined that some of the reported NOAA AR number to HARPNUM associations could not be verified with spatiotemporal co-occurrence analysis. We found, in total, 156 discrepancies in the original HARPNUM to NOAA AR number associations^30^, where for 66 associations the given NOAA AR do not spatially and/or temporally overlap (intersect) with the HARP’s trajectory. For the remaining 90 associations, we discovered co-occurrences with unreported NOAA AR numbers.

After careful visual examination together with our co-occurrence similarity indexes (i.e., *cof* and *μ*_*mindist*_), we manually updated 116 of the 156 individual HARP-to-NOAA associations (66 added and 50 removed). The discrepancies and applied updates are presented in Table S.2 (provided in Supplementary File) along with similarity indices, HARP and NOAA AR lifespans, co-existence and co-occurrence intervals. The full list of 156 discrepancies found are also provided as an addendum with remarks.

### Solar flare reports

The NOAA/GOES observations^32^ measure disk-integrated fluxes between 0.1–0.8 *nm* from the Sun using the X-ray Sensors (XRS). When a sudden, yet persistent, X-ray flux increase is detected, the event is flagged as a likely flare. Manual review is performed by NOAA forecasters to produce the final NOAA flare list. The GOES satellites are subject to eclipses by the Earth in the spring and fall, leading to interruptions (blackouts) in the X-ray flux record lasting from minutes to one hour. The background X-ray radiation emitted by the Sun is usually at the level of A- or B-class flares, making it difficult to capture all flares of these classes during higher-activity phases of the solar cycle. C-, M- and X-class flares, on the other hand, are seldom missed, except in periods of intense activity, when the background may even exceed C1.0. Figure 2 presents an example GOES X-ray flux series annotated with some flare occurrences. As data from the XRS has no spatial information, NOAA uses data from the Solar X-ray Imager (SXI) on the same GOES satellites^33^, which captures full-disk images with one-minute cadence in filter bands ranging from 0.6 to 6 *nm*, as well as other data sources, aiming to pin-point each flare location.

This spatiotemporal information on solar flares allows NOAA’s Space Weather Prediction Center (SWPC) to co-locate the active region responsible for a given flare. Nonetheless, the GOES catalog is not perfect: the locations and NOAA active region numbers are missing for many B-, C- and even a few M-class flares. Our goal is to create a set of clean, cross-checked flare reports. Therefore, we integrated the centroid locations of NOAA ARs to GOES flare reports without an explicit spatial location (i.e., only the NOAA AR numbers are listed), and later cross-checked these locations with two independent feature reporting modules, SSW Latest Events^27^ and Hinode-XRT^29^. Hereafter, we will refer to flare reports from SSW Latest Events and Hinode-XRT modules as SSW and XRT flares, respectively.

#### Data acquisition

We considered the GOES flare catalog as our primary data source. We then used SSW and XRT flares along with NOAA AR locations to enhance, verify, and clean the data. The GOES flare reports were downloaded using SunPy modules^34^, which obtain data from HEK. The SSW flares were downloaded directly from their web archive^27^, due to the inconsistencies between the web archive and HEK records. The XRT flares were downloaded directly from the online XRT Flare Catalog^29^. Additionally, we downloaded the 1-minute averaged GOES X-ray flux (0.1 to 0.8 nm) time series available from NOAA, as well as the NOAA AR data from the NOAA Solar Region Summary (SRS)^23^.

During the period of interest that spans more than eight years (2010-05-01 to 2018-12-31), there are 14,401 GOES flare records, distributed into 50 X-, 742 M-, 7,754 C- and 5,817 B-, and 38 A-class events. We also downloaded 14,570 XRT flares and 14,443 SSW flares. All three data sources have the following common attributes: start time, peak time, end time, NOAA active region number, GOES class, and point location (i.e., heliographic latitude and longitude (central meridian distance), in degrees). Additionally, we utilize the daily NOAA active region list, which includes both numbered sunspot and plage regions, totaling about 16,045 daily NOAA active region reports.

#### Data enhancement and verification for GOES flares

We schematically show our flare enhancement and cross-checking procedures in Fig. 3. The first step of the procedure involves a data enrichment process for GOES flares lacking an explicit point location, using their associated NOAA active regions. Then, we attempt to match each GOES flare to an SSW and an XRT flare using the temporal attributes (start and end times of flares) and flare magnitudes. For the GOES flares that we now have location information and matched secondary flare source information, we cross-check the flare locations from these three data sources to verify their authenticity. Lastly, if the GOES flare locations are still missing or could not be verified using the locations from secondary flare data sources, we perform a secondary location augmentation using only the secondary data sources (SSW and XRT).

Among 14,401 GOES flares, only 4,999 have explicit locations and 9,402 do not. For these missing locations we use the associated NOAA active region locations as a proxy. With NOAA active region location augmentation, we determined the approximate locations of 7,104 flares. The vast majority of the remaining 2,298 GOES flares with undetermined locations were A-, B- and C-class flares (2,265 or 98.56% of them). These cases did not have location information or NOAA active region association.

For those GOES flares with original or augmented location information we found the corresponding SSW flare report, which has the same magnitude and is temporally overlapping. In case of multiple candidate SSW flare reports, we picked the spatially closest one to the GOES flare. The same procedure was applied for XRT flares. In the end, for 14,239 (out of 14,401) GOES flares, we found at least one flare report from SSW or XRT flares; and for 12,716 of them we found a flare report from both SSW and XRT flares. Only 162 GOES flares with location information, could not be matched to either SSW or XRT flares.

For each matched flare, we also found the distances among GOES, SSW, and XRT reported coordinates. Namely, we calculated three distances: (1) *d*_*GS*_ – distance between GOES and SSW coordinates, (2) *d*_*GX*_ – distance between GOES and XRT coordinates, and (3) *d*_*SX*_ – distance between SSW and XRT coordinates. We used these distances in our distance-based verification step. An example illustration of distances between GOES-, SSW-, and XRT-reported flare coordinates is shown in Fig. S.3 (provided in Supplementary File). The reported locations from GOES, SSW, and XRT are usually different. While most of these differences are negligible, some are not. There are a variety of reasonable explanations for these differences, including the numerical accuracy of the reported coordinate (i.e., the number of decimal places reported), use of approximate active region location augmentation (both by us and by XRT), or pixel bleeding^35^. However, for the large differences, often times reporting modules either do not report correct coordinates (such as, say, flares at extreme heliographic latitudes) or there are multiple flares occurring close to the solar limbs.

In matching the locations of GOES, SSW and XRT flares, we chose to use 275 *arcsec* (in helioprojective coordinates^36^) as the proximity threshold for distance-based verification. We determined this threshold after a careful examination of M- and X-class flares, which had relatively large distances (>150 arcsec) in their reported locations from different data sources. The reported locations of the examined flares and the notes and links to those flare reports can be found in the additional files of the dataset. We also acknowledge that the coordinate system we use for the verification, Helioprojective Cartesian (HPC), carries a bias for flares and active regions occurring near the limbs, due to foreshortening. This implies that uncertainties in flare locations derived from pixel coordinates will be much higher for flares near the limbs. We used the more inclusive 275 *arcsec* threshold to reduce the possible bias in practice.

In the course of flare verification process, if for a GOES flare there is at least one secondary flare report within 275 arcsec (*d*_*GX*_ < 275 or *d*_*GS*_ < 275), we mark that flare as *primary-verified*. If this is not the case, but the distance between the SSW and XRT reported locations is less than 275 arcsec (*d*_*SX*_ < 275), we mark it as *secondary-verified*. For secondary-verified flares, the reported GOES location is not close to either of the SSW or XRT locations; however, SSW and XRT locations are in agreement. If a flare is neither primary- nor secondary-verified, we mark it as *non-verified*. If a flare is marked as verified, either primary or secondary, it means that its existence is confirmed with at least two independent observations and detections. Note that the need for a secondary-verification step using NOAA AR location information could be an artifact of our GOES flare location augmentation. Although the use of the NOAA AR interpolated center is a suitable way to assign flare locations, a flare could easily have occurred near the edge of the NOAA AR as opposed to its interpolated center location. Our threshold of 275 arcsec corresponds roughly to the linear dimensions of a sizable active region.

We present the distributions of the minimum distances between either GOES and SSW or GOES and XRT reported locations in Fig. S.4 (in Supplementary File). It can be seen that the vast majority of the ≥*M*1.0 flares have distance smaller than 150 arcsec between the GOES location and the secondary location (from either SSW or XRT). We also present the heatmaps of the minimum distance used for verification for different classes of flares for both primary- and secondary-verified in Fig. S.5 (in Supplementary File). The relatively higher distances (>150 arcsec) between primary and secondary locations are scattered across the disk. Thus, we can claim that the intrinsic bias of the HPC coordinate system close to the solar limbs is not propagated to the data.

The last step of our flare enhancement procedure is the augmentation of the flare record with the secondary flare locations. For each secondary-verified flare, the GOES reported location is replaced with the XRT location, while the XRT locations are verified using the SSW locations. The latitudes of primary-verified, secondary-verified, and non-verified flares over time are shown in Fig. S.6(a–c), respectively (in Supplementary File). We notice a concentration of non-verified flares over the second half of 2010, when the SSW Latest Events module was not operating. Naturally, then, this period does not include any secondary verified flares. We also see a few clusters in non-verified flares which correspond to outages of the SDO/AIA instrument.

#### Resulting flares

After the NOAA AR augmentation and flare cross-checking steps, between May 1 2010 and December 31, 2018 we have 10,878 primary verified, 2,763 secondary verified, and 760 non-verified GOES flares. There are 50 X-class, 730 M-class, 7,556 C-class, and 5,305 A- or B-class flares that were verified (primary or secondary). All X-class flares are primary verified. Only 12 out of 742 M-class (1.6%) are not verified. About 97.4% of C-class flares and 90.6% of A- and B-class flares are verified. Given their small size and abundance, the majority of non-verified flares are, therefore, A- and B-class events.

**Fig. 4:**
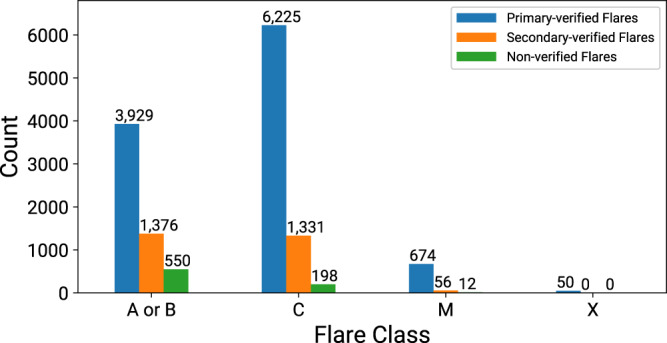
Scatter plot of the primary- and secondary-verified heliographic latitudes of flares (in degrees), as a function of peak times, ranging between May 1, 2010 and December 31, 2018.

**Fig. 5:**
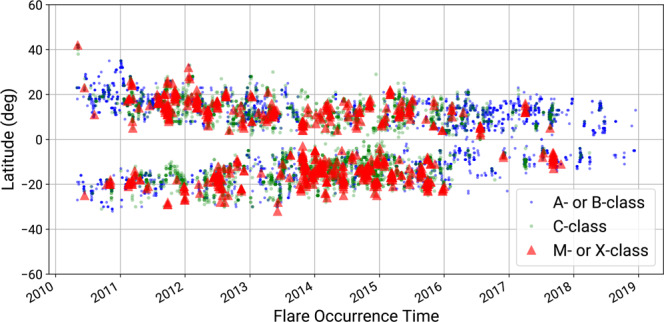
The number of flares for each GOES flare class after flare cross-checking procedures were applied. Blue bars show the primary-verified flares, with cross-checked GOES locations, orange bars show the secondary-verified flares whose GOES location could not be verified and green bars show the non-verified flares.

Figure 4 shows histograms of verified and non-verified flares per flare class, while Fig. S.7 (in Supplementary File) shows the spatial distribution of verified and non-verified flares. Figure 5 depicts the scatter plot of verified flare latitudes and peak times as a function of time, which is reminiscent of the long known butterfly diagram for sunspots^37^.

**Fig. 6:**
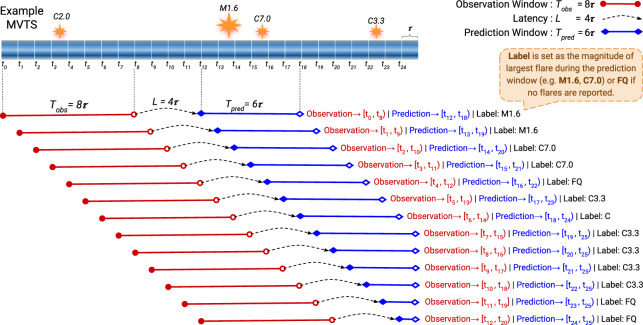
Example slicing and labeling of time series, characterized by an elementary time unit of length $\tau$. Time steps (*t*_*i*_) can then be defined at instances corresponding to integer multiples of $\tau$.

### SHARP data and magnetic field parameters

A HARP data collection (and the corresponding SHARPs) consist of a 12-minute sampled time series of spatial cutouts including the vector magnetic field, continuum intensity, and maps or values of other quantities. Each HARP may contain one or more solar active regions within the cutout region. Each HARP series is labeled with a unique identifier, HARPNUM. The number of observations in HARP series depends on how long the active region(s) it encloses were visible on the solar disk.

There are two types of HARPs (and associated SHARP metadata) available from JSOC: the *definitive* and the *near real-time* (*NRT*). The NRT series is useful for space weather forecasting in an operational context as it is processed within three hours of acquisition. However, the NRT dataset pipeline changes the bounding box size of HARPs as they evolve and assigns different identifiers to active regions within the series that might merge or split as they traverse the disk. This makes it difficult to associate flares to specific HARPs and this is why we have chosen to utilize the definitive series instead.

The definitive series is processed after observing a HARP for its entire rotation across the earthward solar hemisphere. A maximal bounding box, which can often encompasses multiple active regions within a HARP is chosen and remains fixed in this case. Active regions that merge or split are also tracked as a single, all-encompassing HARP. The higher data quality and consistency makes the definitive series a better option for creating benchmark datasets that increase our physical understanding of space weather phenomena and their possible links to the photospheric magnetic field, including the identification and optimization of solar flare predictors.

HARP magnetogram time series are available in two coordinate systems: native CCD and Lambert cylindrical equal area (CEA^36^). In the CEA projection, the vector magnetic field is decomposed into radial (*r*), westward ($\phi$), and southward (*θ*) components. This projection is very convenient for calculating various extensive (i.e., area- or size-dependent) quantities, such as the total area of the active region, its magnetic flux, etc. For our dataset, we have used the definitive series mapped to CEA projection with 720 seconds cadence (hmi.Sharp_cea_720s). Provided that this dataset results in improved flare forecasting performance, the next step will be the creation of an NRT dataset for the pre-operational testing of prediction algorithms. Any performance discrepancies between the two series could then be attributed to caveats and shortcomings of the NRT dataset.

#### Magnetic field parameters

It has become generally accepted that, since flares are predominantly magnetic phenomena, a viable flare forecast could rely on the choice of adequate magnetic field properties and prediction methods (see, e.g.^21,38–42^). Therefore, we use the definitive hmi.Sharp_cea_720s data series to calculate the parameters discussed in^21^ using the vector magnetic field. We have chosen to recalculate these parameters ourselves to, first, validate these data and achieve better maintainability and, second, complement them with parameters not currently present in SHARP headers but considered important for flare and coronal mass ejection prediction.

We emphasize that many, but by no means all, of the existing flare-prediction studies did not consider these magnetic field parameters as time series. Instead, forecasting relied on cross-sectional, or point-in-time (snapshot) parameter values^42–44^. There are a few exceptions: Gallagher *et al*.^45^, Falconer *et al*.^46^, and Leka *et al*.^47^ used the rate or previous flaring in an active region. Leka *et al*.^47^ also derived two coefficients (slope and intercept of a linear fit) of flare-predictive time series parameters. Lee *et al*.^48^ used the temporal change in active region area and McCloskey *et al*.^49^ considered the evolution of sunspot characteristics as a flare predictor. Boucheron *et al*.^50^ considered time evolution parameters for predicting the flare size and time-to-flare.

To facilitate both point-in-time and time series analysis, we derive a set of magnetic field parameters from individual region patches and transform them into multivariate time series over the entire length of a given HARP series. This way we enable the analysis of the active region evolution by systematically analyzing high-cadence time series for the parameters we calculate. Full time series, second-order moments thereof, as well as point-in-time values chosen within these time series, for any given physical parameter, are then fully enabled for prediction. To our knowledge, this avenue has yet to be systematically investigated for space weather prediction and we believe it will be promising for this purpose.

A number of physically important and potentially flare-predictive magnetic field parameters have been listed by^21^ and are reproduced in Table 1. However, as previously mentioned, several of our MVTS parameters (marked with an asterisk in Table 1) are not included in the original SHARP header information. For the generation of these parameters, we used the following information: *B*_*r*_ (radial component of the magnetic field), *B*_*θ*_ (southward/poloidal component of the magnetic field), ${B}_{\phi }$ (westward/toroidal component of the magnetic field), *BITMAP* (active region boundary), *MAGNETOGRAM* (line of sight magnetogram), and *CONF_DISAMB* (confidence map of magnetic field disambiguation). Using these segments as inputs to our magnetic field parameter calculation module^51^, we generated time series of all magnetic field parameters listed in Table 1. These recalculated parameters were then compared against the SHARP keyword values for correctness. Note that, as was discussed in^22^, there are daily variations of the radial velocity of the spacecraft inherent to its geosynchronous orbit, which can introduce periodicities in some of the parameters^10^. As our calculations are based on the work of^10^, our recalculated values unavoidably exhibit the same variations that were discussed in that work.

**Table 1:** Computed magnetic field parameters.

Magnetic Field Parameters from^21^	Description	Formula
ABSNJZH^56^	Absolute value of the net current helicity in G2/m	${H}_{{c}_{abs}}\propto \left|\sum {B}_{z}\cdot {J}_{z}\right|$
EPSX*^57^	Sum of X-component of normalized Lorentz force	$\delta {F}_{x}\propto \frac{\sum {B}_{x}{B}_{z}}{\sum {B}^{2}}$
EPSY*^57^	Sum of Y-component of normalized Lorentz force	$\delta {F}_{y}\propto \frac{-\sum {B}_{y}{B}_{z}}{\sum {B}^{2}}$
EPSZ*^57^	Sum of Z-component of normalized Lorentz force	$\delta {F}_{z}\propto \frac{\sum ({B}_{x}^{2}+{B}_{y}^{2}-{B}_{z}^{2})}{\sum {B}^{2}}$
MEANALP^58^	Mean twist parameter	${\alpha }_{total}\propto \frac{\sum {J}_{z}\cdot {B}_{z}}{\sum {B}_{z}^{2}}$
MEANGAM^56^	Mean inclination angle	$\bar{\gamma }=\frac{1}{N}\sum {\rm{\arctan }}\left(\frac{{B}_{h}}{{B}_{z}}\right)$
MEANGBH^56^	Mean value of the horizontal field gradient	$\bar{\nabla {B}_{h}}=\frac{1}{N}\sum \sqrt{\left(\frac{\partial {B}_{h}}{\partial x}+\frac{\partial {B}_{h}}{\partial y}\right)}$
MEANGBT^56^	Mean value of the total field gradient	$\bar{|{\rm{\nabla }}{B}_{tot}|}=\frac{1}{N}\sum \sqrt{\left(\frac{{\rm{\partial }}B}{{\rm{\partial }}x}+\frac{{\rm{\partial }}B}{{\rm{\partial }}y}\right)}$
MEANGBZ^56^	Mean value of the vertical field gradient	$\bar{\nabla {B}_{z}}=\frac{1}{N}\sum \sqrt{\left(\frac{\partial {B}_{z}}{\partial x}+\frac{\partial {B}_{z}}{\partial y}\right)}$
MEANJZD^56^	Mean vertical current density	$\bar{{J}_{z}}\propto \frac{1}{N}\sum \left(\frac{\partial {B}_{y}}{\partial x}-\frac{\partial {B}_{x}}{\partial y}\right)$
MEANJZH^56^	Mean current helicity	$\bar{{H}_{c}}\propto \frac{1}{N}\sum {B}_{z}\cdot {J}_{z}$
MEANPOT^59^	Mean photospheric excess magnetic energy density	$\bar{\rho }\propto \frac{1}{N}\sum {\left({{\boldsymbol{B}}}^{Obs}-{{\boldsymbol{B}}}^{Pot}\right)}^{2}$
MEANSHR^59^	Mean shear angle	$\bar{\Gamma }=\frac{1}{N}\sum {\rm{\arccos }}\left(\frac{{{\boldsymbol{B}}}^{Obs}\cdot {{\boldsymbol{B}}}^{Pot}}{| {B}^{Obs}| | {B}^{Pot}| }\right)$
R_VALUE*^60^	Total unsigned flux around high gradient polarity inversion lines using the *B*_*los*_ component	Φ = Σ$| {B}_{los}| .dA\,(within\,R\,mask)$
SAVNCPP^56^	Sum of the absolute value of the net current per polarity	${J}_{{z}_{sum}}\propto \left|\sum ^{{B}_{z}^{+}}{J}_{z}dA\right|+\left|\sum ^{{B}_{z}^{-}}{J}_{z}dA\right|$
SHRGT45^56^	Area with shear angle greater than 45 degrees	$\frac{{\rm{Area}}\,{\rm{with}}\,{\rm{Shear}} > 4{5}^{\circ }}{{\rm{Total}}\,{\rm{Area}}}$
TOTBSQ*^57^	Total magnitude of Lorentz force	$F\propto \sum {B}^{2}$
TOTFX*^57^	Sum of X-component of Lorentz force	${F}_{x}\propto \sum {B}_{x}{B}_{z}dA$
TOTFY*^57^	Sum of Y-component of Lorentz force	${F}_{y}\propto \sum {B}_{y}{B}_{z}dA$
TOTFZ*^57^	Sum of Z-component of Lorentz force	${F}_{z}\propto \sum ({B}_{x}^{2}+{B}_{y}^{2}-{B}_{z}^{2})dA$
TOTPOT^56^	Total photospheric magnetic energy density	${\rho }_{tot}\propto \sum {\left({\overrightarrow{{\boldsymbol{B}}}}^{Obs}-{\overrightarrow{{\boldsymbol{B}}}}^{Pot}\right)}^{2}dA$
TOTUSJH^56^	Total unsigned current helicity	${H}_{{c}_{total}}\propto \sum {B}_{z}\cdot {J}_{z}$
TOTUSJZ^56^	Total unsigned vertical current	${J}_{{z}_{total}}=\sum | {J}_{z}| dA$
USFLUX^56^	Total unsigned flux in Maxwells	Φ = $\sum | {B}_{z}| dA$

Parameters marked with asteriks (*) are discussed in^21^, but are not available in SHARP headers.

**Table 2:** Summary and categorization of the time series parameters in our dataset.

Parameter Category	Time and Location	Magnetic Field Parameters (Table 1)		Flare History Parameters		Quality
Individual Parameters		ABSNJZH	EPSX			
		EPSY	EPSZ			
	TIMESTAMP	MEANALP	MEANGAM	BFLARE	BFLARE_LABEL^a^	QUALITY
	LAT_MIN	MEANGBH	MEANGBT	BFLARE_LOC	BFLARE_LABEL_LOC^a^	XRQUALITY^b^
	LON_MIN	MEANGBZ	MEANJZD	CFLARE	CFLARE_LABEL^a^	CRVAL1
	LAT_MAX	MEANJZH	MEANPOT	CFLARE_LOC	CFLARE_LABEL_LOC^a^	CRVAL2
	LON_MAX	MEANSHR	SAVNCPP	MFLARE	MFLARE_LABEL^a^	CRLN_OBS
	HC_ANGLE	SHRGT45	TOTBSQ	MFLARE_LOC	MFLARE_LABEL_LOC^a^	CRLT_OBS
	NOAA_AR	TOTFX	TOTFY	XFLARE	XFLARE_LABEL^a^	SPEI
		TOTFZ	TOTPOT	XFLARE_LOC	XFLARE_LABEL_LOC^a^	IS_TMFI
		TOTUSJH	TOTUSJZ	XR_MAX^b^		
		USFLUX	R_VALUE			

^a^The flare label series (e.g., CFLARE_LABEL or XFLARE_LABEL_LOC) are stored as annotations in the form of JSON objects, shown as follows:

{

“magnitude” : [GOES class of the flare],

“id” : [flare identifier],

“NOAA_AR” : [associated NOAA active region number if available],

“narn_source” : [data source where NOAA_AR is obtained- GOES, SSW, or XRT]

“verification” : [verification flag- Primary, Secondary, or Non-verified]

}.

^b^XR_MAX series signifies the maximum X-ray flux (from 1–8 Angstrom), while XRQUALITY is the quality flag showing its quality.

#### Cleaning the MVTS

The cleaning steps we took in our MVTS account for empty SHARPs, location-based filtering, and missing values. Firstly, we removed the empty SHARPs, which possibly resulted due to post-processing merging of NRT HARPs. After this, we recovered 4,098 MVTS files representing over 520,000 hours of solar activity. Furthermore, about 8.34% of timestamps were missing in the time series and were filled with *null* values to maintain a fixed cadence of 12 minutes. Potential reasons for these data gaps are, first, gaps in the SHARP series when the HARP is close to the eastern solar limb or when it is about to rotate beyond the western limb and, second, the SDO eclipse seasons.

To warn about severe projection effects and the low signal-to-noise ratio for magnetic field measurements near solar limbs, while still allowing the interested researchers to perform limb-to-limb analyses, we added a Boolean flag, *TMFI* (trusted magnetic field information) to our MVTS dataset. TMFI was set to True for regions with (1) CMD within 70 degrees from the solar disk center and (2) SHARP *QUALITY* index equal to zero. A non-zero *QUALITY*^10^ value in the SHARP header corresponds to magnetic field observables created under sub-optimal conditions and hence these records are flagged as not trustworthy by setting *TMFI* as False.

### Flare integration with SHARP data

The NOAA/GOES flare reports have three temporal attributes (start, peak and end times) and two spatial attributes, namely the explicit coordinate location and implicit NOAA AR number. Moreover, as the HARP detection module identifies smaller active regions and reorganizes the reports for the definitive series, HARPNUMs (identifiers of HARP series) do not show a one-to-one correspondence with NOAA AR numbers. There are some SHARP series not mapped to any NOAA ARs, while others are mapped to multiple NOAA ARs. The list of HARPNUM to NOAA AR number associations are provided by JSOC^30^. However, we identified a few discrepancies in that matching and updated this list as described earlier.

Due to these inconsistencies between SHARPs and flare reports, we apply two flare integration procedures based on (1) NOAA AR numbers and (2) location attributes. Utilizing the integrated flare information produced by these two methods, we create eight additional time series parameters of flare history for each MVTS (i.e., four flare classes (B, C, M, and X) for each of the two separate procedures (NOAA AR numbers and locations)). The history series signify the identifier, magnitude, and, when available, NOAA AR number of the flares. Values in the flare history series show the number of flares from a particular class occurring in a given 12-minute interval, associated with a particular HARP record. The flare annotations are inserted in the series at the timestamps closest to the flare peak times.

#### Using NOAA active region numbers

We find all NOAA AR numbers that correspond to a given HARPNUM and search the flare reports only for those NOAA ARs. We then create *NOAA AR number-based* flare history series for B-, C-, M-, and X-class flares separately. All associated flares that occur in the HARP’s lifespan are added. If there are no flares for a particular NOAA AR number or if the resulting subset of associated flares did not occur during the lifespan of the respective HARP series, then no flares are integrated.

#### Using location attributes

For each bounding box in the spatiotemporal trajectory of active regions (obtained using *LAT_MIN*, *LON_MIN*, *LAT_MAX*, and *LON_MAX* keywords of SHARP headers), we perform a spatiotemporal search on the flare reports. We initially perform a temporal search for flares that occurred during the lifespan of the SHARP series. Next, for each flare report, we check if its spatial location is within the bounding box of the HARP region at its peak time. The result is a list of flares that spatiotemporally overlap with the SHARP series, and we use these series to create the *location-based* flare history series for B-, C-, M-, and X-class flares.

#### X-ray flux integration

In addition to flare history parameters, we integrate the 1-minute averaged GOES X-ray flux data into our MVTS. As discussed already, many NOAA/GOES satellites have an X-ray sensor (XRS) onboard. The first GOES to have an XRS capable of continuous monitoring was GOES-5 and since then many GOES satellites have been used as NOAA’s primary and secondary sources of solar X-ray flux (Table S.3 provided in Supplementary File). Flying in geostationary orbits, these satellites experience a several week period around each equinox when the Earth (or more rarely the Moon) intercepts the line-of-sight between the satellite and the Sun for periods of minutes up to one hour. The eclipse start times plotted against the duration of data gaps from GOES primary satellite data are shown in Fig. S.8(a) (provided in Supplementary File). The X-ray data from primary satellites has a downtime of 1.43% over the period of our dataset. During these downtimes, data from the secondary satellites was used to fill the missing values, which reduced the downtime to 0.80%. The remaining gaps are shown in Fig. S.8(b).

Due to the different cadence between the 1-minute X-ray flux data and the 12-minute MVTS, we chose to report the maximum X-ray flux during the 12-minute interval centered around the timestamps of MVTS records. We also included a quality flag (*XRQUALITY*) to identify X-ray blackouts and data quality issues, which indicates how many of the X-ray recordings in a particular 12-minute interval are valid. The quality flag ranges between 0, when there is a total blackout and no data are available in the 12-minute interval, and 12, when all of the 1-minute averaged data are present for that time period. It should be noted that while flare reports are specific to particular active regions, the X-ray flux is measured over the entire Sun.

### Task-based dataset generation

Our main data product is 4,098 MVTS of solar active region parameters annotated with a collection of co-occurring flares. Each MVTS is directly and uniquely associated to a SHARP. We now establish a methodology for creating machine-learning-ready time series datasets and provide the source code for generating them. The knowledge discovery process starts with determining the data mining task. The entire process of data handling and preparation should be tailored for the task at hand. Supervised machine learning tasks can be loosely separated into two categories based on the characteristics of the target variables: classification (if the target variable is discrete) and regression (if the target variable is continuous). For the task of dataset generation, we focus on supervised classification based on discrete flare labels.

An important step towards accelerating machine learning-based solar physics analyses is providing benchmark datasets that are cleaned, partitioned, properly sliced and labeled, as well as consistently balanced based on the number and ratio of flaring (minority) class instances across partitions. We have already discussed the cleaning procedures applied and will now review the partitioning, slicing, labeling, and balancing procedures. We would like to note here that we have not applied any data transformation or dimensionality reduction procedures because these procedures are dependent on the task and selected models.

#### Partitioning

The first step in creating a machine learning model is to determine the task, and therefore, to specify the target classes. Target classes are determined using flare intensity threshold criteria. For a common binary classification schema, where M- or X-class flares (≥M1.0) are considered flaring and lower magnitude flares (<M1.0) and flare quiet instances are considered non-flaring, target class specification will use a single threshold value [M1.0]. For creating a 4-class classification schema (e.g., B-class or lower (≤B9.9), C-class (≥C1.0 and ≤C9.9), M-class (≥M1.0 and ≤M9.9), and X-class (≥X1.0)), we can use [C1.0, M1.0, X1.0] as the threshold criteria. Different threshold criteria can be produced for different tasks.

It is important to remember that large flares (M- or X-class), which have the greatest impacts on the space environment and are thus the most commonly targeted in predictive analyses, are scarce. In our dataset, we have 730 M-class flares and only 50 X-class flares, corresponding to a mere ~6.8% of all flare records included in the dataset. Among 4,098 MVTS, only 27 contain X-class flares and 178 have M-class flares, corresponding to a slim ~5% of the total. 3,293 MVTS do not have any flares (including B- or C-class flares).

In machine learning applications the creation of validation datasets is usually performed by holding out parts of datasets one or more times, so that the models can learn from the training sets and generalize on samples they have never seen before. Given this scarcity, we propose a more robust validation strategy for machine learning applications to solar flare prediction: *time-segmented stratification*. Besides scarcity, time-segmented stratification is dictated by possible correlations between different time series segments stemming from the same MVTS.

Our stratification method separates the dataset into unequal time intervals (partitions). These different intervals, however, achieve similar total numbers of major flares (i.e., members of the minority class) in each partition. For example, in a partitioned MVTS with balanced minority class populations, a total of 450 M- and X-class flares split between five partitions will give rise to rough totals of 90 M-/X-class flares per partition.

With this method, we can (1) have non-overlapping time segments in each partition, so that the training and testing samples rely on different MVTS, and (2) preserve the number of minority instances across all partitions as much as possible.

#### Slicing and labeling

The following partitioning is to methodically slice and label MVTS based on a desired prediction scenario. To achieve that, we introduce the *observation window*, *latency*, and *prediction window* concepts. We use the observation window length (*T*_*obs*_) to determine the duration of time series slices for the sampling of predictive parameters. To label each of these slices with the appropriate flare occurrence, we determine the latency (*L*) and prediction window (*T*_*pred*_) lengths. Latency represents the time interval from the issuing of a forecast (end of the observation window) to its coming into effect at the start of the prediction window. The prediction window is then the interval of validity of that forecast. We use *T*_*obs*_, *L*, and *T*_*pred*_ as user-defined input parameters for custom slicing and labeling.

For a time series slice (i.e., observation, latency and prediction windows) starting at *t*_*i*_, the observation window corresponds to the interval at $$[{t}_{i},{t}_{i}+{T}_{obs})$$. The prediction window corresponds to the interval at $$\left[{t}_{i}+{T}_{obs}+L,{t}_{i}+{T}_{obs}+L+{T}_{pred}\right)$$. Each instance (slice) are then labeled with the magnitude of the largest flare (if any) that occurred in that HARP during the prediction window. A schematic, exemplary scenario of slicing and labeling for a MVTS is presented in Fig. 6.

Though optional, another important step is to ensure the quality of the individual slices and their labels. There are three factors that may impact the quality. The first is the lack of trusted magnetic field information, with quality of individual records in slices checked using the TMFI parameter. The second is the lack of high quality X-ray flux data. The slices whose prediction window coincides with a prolonged period of unavailable or low quality X-ray flux data should be eliminated, as possibly missed flare reports during these intervals may mistrain models. This can be checked using the XRQUALITY parameter. The third is the non-verified flare reports, which can result in mislabelings, primarily for non-flaring slices whose prediction window coincides with the peak times of large non-verified flares. For completeness, we provide these non-verified flare reports as an addendum to our dataset.

#### Undersampling for class imbalance

The last step in our dataset generation procedure is adjusting the class distributions, of majority and minority classes, in each partition. Note that the terms, minority and majority, are used in the context of number of occurrences and not energy levels of flares. Despite different frequencies of large flares during different parts of solar cycle, the representation of instances from minority class (usually M- or X-class flares) should be consistently proportional among each time-segmented partition. To achieve this, we can use use different undersampling or oversampling techniques. We provide an example undersampled dataset as addenda to our dataset. A more detailed study on undersampling and oversampling for flare prediction is available in our recent studies^52,53^.

### Extending the datasets

While the dataset generation procedures described here provide a framework for testing the validity of predictions of solar flares, we envision possible directions to extend and improve the dataset. We present two methods of extension, namely a “horizontal” and a “vertical” one.

A horizontal extension would be the addition of more time series variables (parameters) to our dataset. These parameters would add new dimensions to our original dataset in the interest of improving predictions. Possible horizontal extensions include addtional magnetogram-based metadata parameters, measures of photospheric or coronal intensity, the latter for various wavelengths, measures of the Doppler velocity and a horizontal velocity inferred by line-of-sight or vector magnetograms and centered around each 12-minute instance, as well as background X-ray levels or adjacent morphological features such as X-ray sigmoids, filaments, coronal holes, etc. and the distance of the active region location from them.

A vertical extension would be an integration of additional phenomena of space weather interest. These resources, similar to flare reports, could be annotated to enhance the predictive potential of the datasets. Examples of vertical extensions include: CMEs, filament eruptions, or solar energetic particle (SEP) events.

## Data Records

As described throughout this paper, our benchmark dataset MVTS originated from the SHARP data series covering the period from 2010-05-01 to 2018-08-31. The data records along with supplementary data files are available through Harvard Dataverse^54^, along with usage notes. Each of these MVTS consists of 51 parameters (not including timestamps). We categorized these parameters into four groups and listed the individual parameters in each group in Table 2. The *time and location* parameters include timestamp and bounding box information, as well as the corresponding NOAA active region number demonstrating the implicit location of active regions. Location parameters, i.e., LAT_MIN, LON_MIN, LAT_MAX, LON_MAX, show the HARP bounding box locations. NOAA_AR series signifies the corresponding NOAA active region number, when available. The quality parameters include magnetic field and X-ray quality information (XRQUALITY) along with the TMFI flag. Two large groups of parameters are *magnetic field* and *flare history parameters*. Details on magnetic field parameters are demonstrated in Table 1. The flare history parameters show the number of associated flares in the form of time series. Each value (at *t*_*i*_) in these time series shows the number of flares occurred between *t*_*i*_ and (*t*_*i*_ + 12 minutes). BFLARE, CFLARE, MFLARE, XFLARE series signify the flare counts (of particular classes of flares) integrated using NOAA active region numbers, while BFLARE_LOC, CFLARE_LOC, MFLARE_LOC, XFLARE_LOC series are flares integrated using location attributes.

In total, we have 4,098 MVTS in our series. The MVTS files are stored in CSV format and the name of files correspond to the HARPNUM of the SHARP series. Each file stores 51 time series parameters, equidistributed with 12 minute cadence.

## Technical Validation

Our technical validation can be summarized in two courses of action: (1) the comparison of the magnetic field parameters we calculated with those provided in SHARP headers, and (2) the cross-checking of the flare reports we obtained from GOES with the SSW and XRT flares. Our analysis of magnetic field parameters shows consistency with the values reported in SHARP headers, with minimal discrepancies due to minor implementation differences. In particular, our comparisons show that ~96.6% of our calculated values differ by less than 1% and 98.1% of them differ by less than 2% from the SHARP values. Most of the differences (~90% in both cases) between values correspond to the SHRGT45 parameter (Table 1).

Differences between our estimations and SHARP headers in the range 0–1% can be attributed to the double-precision floating point variables that we utilize for calculations. To our knowledge and understanding, JSOC calculations used to provide SHARP headers were performed in single precision. This leads to differences in calculated values from a given algorithm and explains why we used a 1% difference threshold in our validation step. This said, there are cases of differences between our parameter values and SHARP headers that are above it, referring mainly to the SHRGT45 parameter and, secondarily, to the MEANSHR parameter. Discrepancies are due to the fact that we did not include computed uncertainties for the radial (*B*_*r*_), westward (${B}_{\phi }$), and southward (*B*_*θ*_) components of the CEA vector magnetic field. The original calculations for the SHARP headers would not include pixel locations in the SHRGT45 calculation that had a not-a-number (NaN) value in the uncertainty files for any of these components. Therefore, in case of an unknown error at all pixels in the calculation area, SHARP headers produce NaN entries while ours calculate a value, which makes our calculations significantly different in these cases.

The uncertainty files were not included for storage and computational efficiency as well as because the effects of their omission are overall negligible. Cases with non-negligible differences occur almost exclusively close to the limbs (i.e., beyond ±70 degrees from the central meridian), when magnetic field measurements are generally not trusted. Such cases, however, are covered by our TMFI flag.

## Supplementary information

Supplementary Information

## Data Availability

Our open-source repositories for MVTS generation, task-based sampling, and model validation is available on Bitbucket^55^. Interested parties are encouraged to get involved in the ongoing development for and extensions to the dataset.
