# Supplementary material for: Multivariate time series dataset for space weather data analytics
Source: Sci Data. 2020 Jul 10;7:227. doi: 10.1038/s41597-020-0548-x (PMC7351763; doi:10.1038/s41597-020-0548-x)
Supplement: Supplementary file 1 — Supplementary Information [file 41597_2020_548_MOESM1_ESM.pdf]

Supplementary File

List of Figures

S.1 The distribution of daily latitudinal (a) and longitudinal (b) displacement of NOAA active regions (including plages) in the form of box plots. Active regions are grouped based on their average latitudes throughout their lifetime into four groups. Four latitudinal zones in the northern and southern solar hemispheres have been introduced as discussed in the text. 3
S.2 Examples of artifacts in the locations of NOAA ARs 11321, 11490, and 11963 in (a), (b), and (c) respectively. 4
S.3 Reported coordinates of the X3.3 flare occurred on 2013-11-05 at 22:12:00 from GOES, SSW Latest Events and Hinode-XRT. The Euclidean distances between these coordinates are recorded for distance-based verification. Coordinates are reported in Helioprojective Coordinate System. All locations and distances are in arcsec. 18
S.4 The distributions of the minimum distances found between GOES and SSW or XRT-reported locations for primary (a) and secondary (b) verified flares. 18
S.5 The heatmaps of the minimum distances used between GOES and SSW or XRT-reported locations for primary and secondary verified flares. (a) Primary-verified A- and B-class flares. (a) Primary-verified A- and B-class flares. (b) Primary-verified C-class flares. (c) Primary-verified M-class flares. (d) Primary-verified X-class flares. (e) Secondary-verified A- and B-class flares. (f) Secondary-verified C-class flares. (g) Secondary-verified M-class flares. There are no secondary-verified X-class flares in our flare list. 20
S.6 The latitudes of the primary-verified (a), secondary-verified (b), and non-verified (c) flares over time. 21
S.7 The spatial distributions of the primary-verified (a)(i-ii-iii), secondary-verified (b)(i-ii-iii), and non-verified (c)(i-ii-iii) flares over time. For each verification category, M-/X-class flares, C-class flares and A-/B-class flares are shown in plots (i), (ii), and (iii), respectively. The number of flares with no valid coordinates are also annotated in each plot. 22
S.8 (a): Blackout durations in GOES XRS data from primary satellites show a biannual periodicity. Total downtime for the period of our dataset was 1.43%. (b): After filling the gaps in primary GOES XRS data with available data from secondary satellites, the total downtime was reduced to 0.80%. 23

List of Tables

S.1 The list of manually updated NOAA AR trajectories with original and modified locations 5
S.2 116 manual updates (denoted with 'Yes' in 'Updated' column, either as additions to or removals from the original JSOC list) to HARP-to-NOAA associations along with the co-occurrence factor (cof) and average minimum distance ($\mu_{mindist}$ – in degrees). A detailed version of this table with remarks and trajectory lifespans is also available as addenda to the dataset. 14
S.3 Dates and times of primary and secondary NOAA GOES satellite coverage for XRS data. Note that this table has been reproduced only for the period between 2010-05-01 and 2018-09-01, which matches the duration of our dataset. *Data courtesy:* Dr. Janet Machol. 19

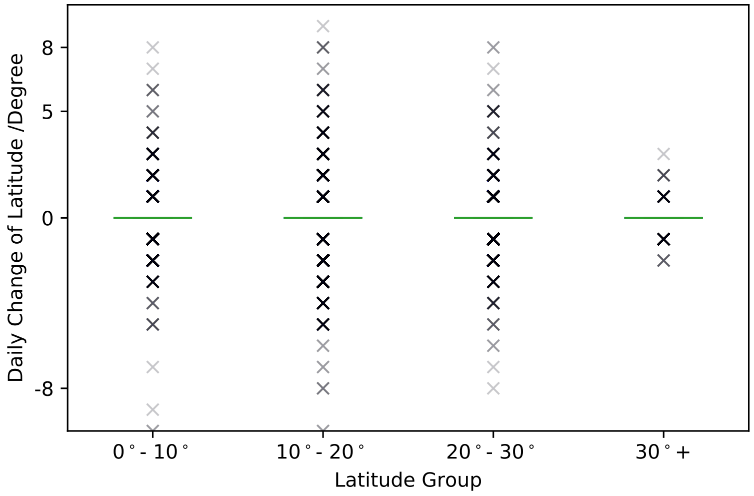

(a)

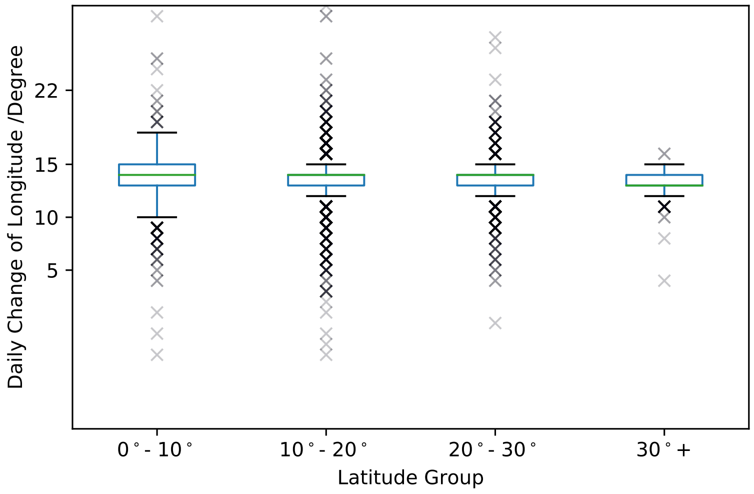

(b)

Figure S.1: The distribution of daily latitudinal (a) and longitudinal (b) displacement of NOAA active regions (including plages) in the form of box plots. Active regions are grouped based on their average latitudes throughout their lifetime into four groups. Four latitudinal zones in the northern and southern solar hemispheres have been introduced as discussed in the text.

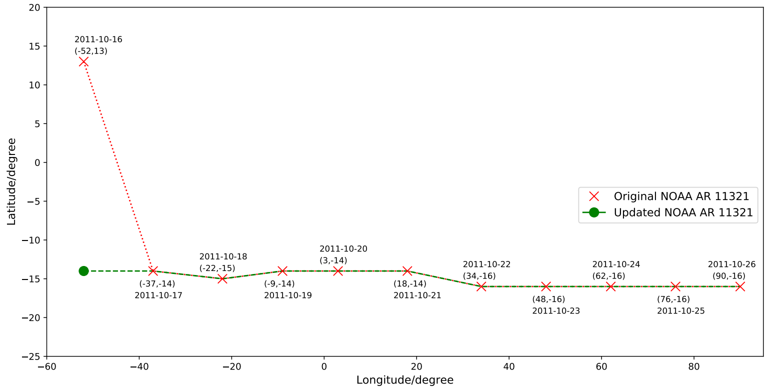

(a)

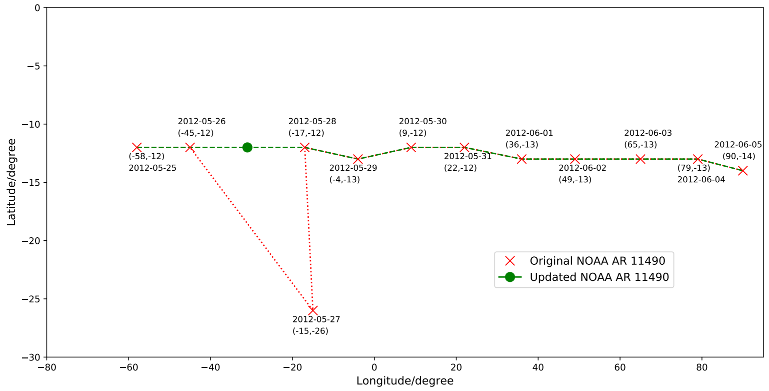

(b)

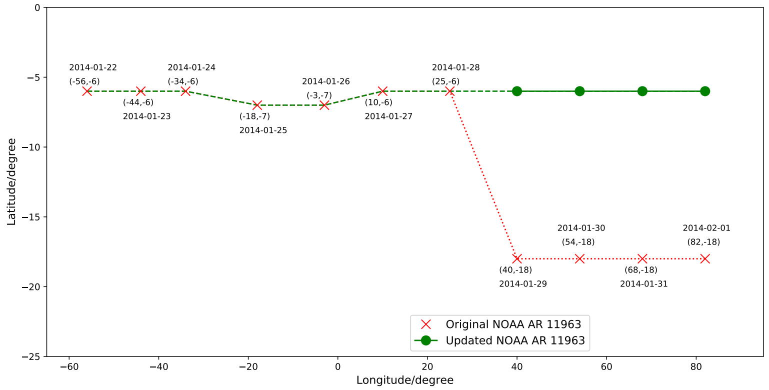

(c)

Figure S.2: Examples of artifacts in the locations of NOAA ARs 11321, 11490, and 11963 in (a), (b), and (c) respectively.

Table S.1: The list of manually updated NOAA AR trajectories with original and modified locations

NOAA AR Number	Year	Month	Day	Longitude	Latitude	Modified Longitude	Modified Latitude
11048	2010	2	14	-90	23	-78	
11048	2010	2	15	-64	20	-70	
11048	2010	2	16	-50	21		
11048	2010	2	17	-34	21		
...	...	...	...	...	20		
...	...	...	...	...	...		
11058	2010	3	27	21	25		
11058	2010	3	28	-73	-21	34	25
11058	2010	3	29	47	25		
11058	2010	3	30	60	25		
...	...	...	...	...	...		
...	...	...	...	...	...		
11072	2010	5	25	22	-15		
11072	2010	5	26	36	-15		
11072	2010	5	27	52	-23		-15
11072	2010	5	28	65	-15		
11072	2010	5	29	79	-15		
...	...	...	...	...	...		
11076	2010	6	3	22	-19		
11076	2010	6	4	28	-27		-19
11076	2010	6	5	49	-19		
11076	2010	6	6	61	-19		
...	...	...	...	...	...		
...	...	...	...	...	...		
11100	2010	8	24	34	-27		
11100	2010	8	25	47	-27		
11100	2010	8	26	64	-19		-23
11100	2010	8	27	78	-20		-23
11100	2010	8	28	91	-20		-23
...	...	...	...	...	...		
11108	2010	9	21	-24	-30		
11108	2010	9	22	-14	-30		
11108	2010	9	23	9	-30	4	
11108	2010	9	24	22	-30		
11108	2010	9	25	34	-30		
...	...	...	...	...	...		
...	...	...	...	...	...		
11120	2010	11	5	-4	38		
11120	2010	11	6	9	38		
11120	2010	11	7	13	41	17	

11120	2010	11	8	26	41		
11120	2010	11	9	39	41		
...	...	...	...	...	...		
...	...	...	...	...	...		
11121	2010	11	11	1	-20		
11121	2010	11	12	28	-23	21	
11121	2010	11	13	41	-23		
11121	2010	11	14	54	-23		
...	...	...	...	...	...		
...	...	...	...	...	...		
11125	2010	11	15	6	18		
11125	2010	11	16	21	18		
11125	2010	11	17	43	20	38	
11125	2010	11	18	56	20		
...	...	...	...	...	...		
...	...	...	...	...	...		
11150	2011	2	4	13	-22		
11150	2011	2	5	30	-20		
11150	2011	2	6	46	-20	41	
11150	2011	2	7	51	-22	53	
11150	2011	2	8	63	-23		
11150	2011	2	9	77	-23		
...	...	...	...	...	...		
11163	2011	3	7	65	17		
11163	2011	3	8	79	17		
11163	2011	3	10	89	-26	Remove	Remove
...	...	...	...	...	...		
11181	2011	3	31	-6	-26		
11181	2011	4	1	8	-27		
11181	2011	4	2	22	-27	20	
11181	2011	4	3	36	-27	29	
11181	2011	4	4	50	-27	41	
11181	2011	4	5	50	-27	57	
11181	2011	4	6	64	-27		
11181	2011	4	7	78	-27		
...	...	...	...	...	...		
11185	2011	4	8	-24	24		
11185	2011	4	9	-14	20		
11185	2011	4	10	-15	18	-3	
11185	2011	4	11	8	17		
11185	2011	4	12	21	17		
...	...	...	...	...	...		
...	...	...	...	...	...		
11222	2011	5	29	66	16		
11222	2011	5	30	80	16		

11222	2011	5	31	83	16	89	
11222	2011	6	1	98	17		
11228	2011	5	30	-67	17		
11228	2011	5	31	-55	17		
11228	2011	6	1	-50	17	-43	
11228	2011	6	2	-31	17		
11228	2011	6	3	-20	17		
...	...	...	...	...	...		
11284	2011	8	31	57	-19		
11284	2011	9	1	62	-17	69	
11284	2011	9	2	82	-18		
11284	2011	9	3	96	-18		
11321	2011	10	16	-52	13		-14
11321	2011	10	17	-37	-14		
11321	2011	10	18	-22	-15		
...	...	...	...	...	...		
...	...	...	...	...	...		
11341	2011	11	8	-55	10		
11341	2011	11	9	-41	11		
11341	2011	11	10	-28	18		9
11341	2011	11	11	-14	8		
11341	2011	11	12	-2	8		
...	...	...	...	...	...		
...	...	...	...	...	...		
11342	2011	11	9	-34	18		
11342	2011	11	10	-22	17		
11342	2011	11	11	-15	15	-7	
11342	2011	11	12	10	16	7	
11342	2011	11	13	22	15		
11342	2011	11	14	34	16		
...	...	...	...	...	...		
11347	2011	11	14	-53	7		
11347	2011	11	15	-28	8	-34	
11347	2011	11	16	-14	8		
11347	2011	11	17	-4	9		
...	...	...	...	...	...		
11360	2011	11	26	-17	17		
11360	2011	11	27	-3	17		
11360	2011	11	28	10	17		
11360	2011	11	29	32	17	27	
11360	2011	11	30	45	17		
...	...	...	...	...	...		
...	...	...	...	...	...		
11365	2011	12	7	38	21		
11365	2011	12	8	48	17		

11365	2011	12	9	77	16	62	
11365	2011	12	10	91	16	75	
11367	2011	12	5	-36	-18		
11367	2011	12	6	-22	-18		
11367	2011	12	7	-9	-18		
11367	2011	12	8	3	-26		-18
11367	2011	12	9	15	-26		-18
11367	2011	12	10	33	-18		
...	...	...	...	...	...		
11409	2012	1	22	-50	16		
11409	2012	1	23	-40	19		16
11409	2012	1	24	-27	9		17
11409	2012	1	25	-14	18		
11409	2012	1	26	0	18		
...	...	...	...	...	...		
...	...	...	...	...	...		
11410	2012	1	29	-38	16		
11410	2012	1	30	-37	19	-29	
11410	2012	1	31	-20	20		
11410	2012	2	1	-11	18		
...	...	...	...	...	...		
11420	2012	2	13	-69	13	-73	9
11420	2012	2	14	-64	9	-64	
11420	2012	2	15	-48	13		10
11420	2012	2	16	-36	11		
11420	2012	2	17	-23	10		
...	...	...	...	...	...		
...	...	...	...	...	...		
11432	2012	3	18	39	14		
11432	2012	3	19	53	14		
11432	2012	3	20	68	22		14
11432	2012	3	21	78	14		
11476	2012	5	6	-67	9	-77	
11476	2012	5	7	-63	10	-63	
11476	2012	5	8	-48	10		
11476	2012	5	9	-35	11		
...	...	...	...	...	...		
...	...	...	...	...	...		
11489	2012	5	26	-12	-30		
11489	2012	5	27	2	-30		
11489	2012	5	28	-15	-30	11	
11489	2012	5	29	30	-30	27	
11489	2012	5	30	44	-30		
11489	2012	5	31	58	-30		
...	...	...	...	...	...		

11490	2012	5	25	-58	-12		
11490	2012	5	26	-45	-12		
11490	2012	5	27	-15	-26	-31	-12
11490	2012	5	28	-17	-12		
11490	2012	5	29	-4	-13		
...	...	...	...	...	...		
11492	2012	5	26	-65	-13	-72	
11492	2012	5	27	-31	-40	-59	-13
11492	2012	5	28	-43	-14		
11492	2012	5	29	-30	-12		
...	...	...	...	...	...		
11494	2012	6	1	-69	-16	-78	
11494	2012	6	2	-56	-16	-63	
11494	2012	6	3	-20	-15	-48	
11494	2012	6	4	-36	-16	-32	
11494	2012	6	5	-18	-16		
11494	2012	6	6	-6	-17		
...	...	...	...	...	...		
11495	2012	6	1	-19	-12		
11495	2012	6	2	-6	-14		
11495	2012	6	3	-20	-14	7	
11495	2012	6	4	21	-15		
11495	2012	6	5	32	-15		
...	...	...	...	...	...		
11553	2012	8	24	-50	-23	-58	
11553	2012	8	25	-46	-22	-42	
11553	2012	8	26	-33	-21		
11553	2012	8	27	-18	-21		
...	...	...	...	...	...		
11610	2012	11	8	-55	-22		
11610	2012	11	9	-36	-24	-43	
11610	2012	11	10	-32	-23	-26	
11610	2012	11	11	-15	-23		
11610	2012	11	12	-2	-25		
...	...	...	...	...	...		
...	...	...	...	...	...		
11636	2012	12	28	-30	9		
11636	2012	12	29	-16	9		
11636	2012	12	30	-2	9	-5	
11636	2012	12	31	11	9	9	11
11636	2013	1	1	13	14	18	
11636	2013	1	2	27	14	29	
11636	2013	1	3	41	14		
11636	2013	1	4	55	14		

...	...	...	...	...	...		
11661	2013	1	23	-60	13	-65	
11661	2013	1	24	-56	13	-46	14
11661	2013	1	25	-33	17		
11661	2013	1	26	-20	15		
...	...	...	...	...	...		
...	...	...	...	...	...		
11676	2013	2	22	-13	-19		
11676	2013	2	23	-1	-19		
11676	2013	2	24	20	-13	12	-19
11676	2013	2	25	23	-13	24	-16
11676	2013	2	26	39	-18		
11676	2013	2	27	53	-18		
...	...	...	...	...	...		
11687	2013	3	4	-65	6		
11687	2013	3	5	-50	6		
11687	2013	3	6	-46	10	-39	
11687	2013	3	7	-32	9	-29	
11687	2013	3	8	-18	8		
11687	2013	3	9	-5	8		
...	...	...	...	...	...		
...	...	...	...	...	...		
11726	2013	4	24	49	13		
11726	2013	4	25	63	13		
11726	2013	4	26	85	13	78	
11726	2013	4	27	93	13		
...	...	...	...	...	...		
11738	2013	5	11	33	17		
11738	2013	5	12	43	17		
11738	2013	5	13	59	17	59	
11738	2013	5	14	62	20	71	17
11738	2013	5	15	76	20	82	19
11738	2013	5	16	90	20		
11744	2013	5	10	-61	5	-68	
11744	2013	5	11	-56	4	-56	
11744	2013	5	12	-42	5		
11744	2013	5	13	-28	5		
...	...	...	...	...	...		
...	...	...	...	...	...		
11789	2013	7	10	5	-23		
11789	2013	7	11	19	-23		
11789	2013	7	12	24	-28	32	-24
11789	2013	7	13	37	-27	44	-25
11789	2013	7	14	51	-27	57	-25
11789	2013	7	15	65	-27	76	

11789	2013	7	16	79	-27	89	
...	...	...	...	...	...		
11828	2013	8	24	-14	15		
11828	2013	8	25	0	15		
11828	2013	8	26	13	5		15
11828	2013	8	27	26	5		15
11828	2013	8	28	40	5	39	15
11828	2013	8	29	54	5	51	15
11828	2013	8	30	68	5	64	15
11828	2013	8	31	83	5	82	15
11853	2013	9	27	80	19	76	
11853	2013	9	28	77	19	85	
11853	2013	9	29	91	19		
...	...	...	...	...	...		
11923	2013	12	15	23	17		
11923	2013	12	16	37	17		
11923	2013	12	17	49	1		17
11923	2013	12	18	64	1		17
11923	2013	12	19	79	1		17
...	...	...	...	...	...		
11963	2014	1	27	10	-6		
11963	2014	1	28	25	-6		
11963	2014	1	29	40	-18		-6
11963	2014	1	30	54	-18		-6
11963	2014	1	31	68	-18		-6
11963	2014	2	1	82	-18		-6
...	...	...	...	...	...		
11973	2014	2	12	18	4		
11973	2014	2	13	32	4		
11973	2014	2	14	43	16		5
11973	2014	2	15	57	6		
...	...	...	...	...	...		
...	...	...	...	...	...		
12008	2014	3	19	-31	-11		
12008	2014	3	20	-17	-11		
12008	2014	3	21	-3	-11	2	
12008	2014	3	22	19	-10	17	
12008	2014	3	23	30	-11		
12008	2014	3	24	47	-7		
...	...	...	...	...	...		
...	...	...	...	...	...		
12093	2014	6	25	46	-9		
12093	2014	6	26	53	-11		
12093	2014	6	27	77	-3	66	-10
...	...	...	...	...	...		

12094	2014	6	25	37	-18		
12094	2014	6	26	51	-18		
12094	2014	6	27	68	11	62	-18
12094	2014	6	28	82	-18	79	
12166	2014	9	13	-66	12	-72	
12166	2014	9	14	-61	13	-57	
12166	2014	9	15	-44	13		
12166	2014	9	16	-28	12		
...	...	...	...	...	...		
...	...	...	...	...	...		
12184	2014	10	6	-39	-17		
12184	2014	10	7	-26	-17		
12184	2014	10	8	-22	-23	-17	-18
12184	2014	10	9	-7	-21		
12184	2014	10	10	7	-21		
12184	2014	10	11	12	-13	24	-20
12184	2014	10	12	41	-17		
12184	2014	10	13	54	-17		
...	...	...	...	...	...		
...	...	...	...	...	...		
12277	2015	2	4	6	9		
12277	2015	2	5	18	8		
12277	2015	2	6	32	-8		8
12277	2015	2	7	44	9		
12277	2015	2	8	57	9		
...	...	...	...	...	...		
12282	2015	2	8	-76	15	-81	
12282	2015	2	9	-65	15	-68	
12282	2015	2	10	-51	14	-57	
12282	2015	2	11	-48	11	-48	
12282	2015	2	12	-31	11		
12282	2015	2	13	-16	10		
...	...	...	...	...	...		
12302	2015	3	18	54	10		
12302	2015	3	19	79	10	67	
12302	2015	3	20	84	12	81	
...	...	...	...	...	...		
12312	2015	4	1	43	8		
12312	2015	4	2	58	8		
12312	2015	4	3	55	9	71	
12312	2015	4	4	84	9		
12464	2015	12	6	-68	-12		
12464	2015	12	7	-53	-12		
12464	2015	12	8	-39	-12	-42	
12464	2015	12	9	-18	-12	-30	

12464	2015	12	10	-13	-12	-17	
12464	2015	12	11	-3	-10		
12464	2015	12	12	10	-10		
...	...	...	...	...	...		
12511	2016	3	3	-57	5	-46	
12511	2016	3	4	-32	6	-32	
12511	2016	3	5	-19	6		
12511	2016	3	6	-4	6		
...	...	...	...	...	...		
12574	2016	8	8	-72	8		
12574	2016	8	9	-65	14		6
12574	2016	8	10	-49	5		
12574	2016	8	11	-35	5		
...	...	...	...	...	...		
...	...	...	...	...	...		
12683	2017	9	28	-46	13		
12683	2017	9	29	-26	14	-31	
12683	2017	9	30	-21	14	-19	
12683	2017	10	1	-8	13		
...	...	...	...	...	...		

Table S.2: 116 manual updates (denoted with 'Yes' in 'Updated' column, either as additions to or removals from the original JSOC list) to HARP-to-NOAA associations along with the co-occurrence factor (cof) and average minimum distance ($\mu_{mindist}$ – in degrees). A detailed version of this table with remarks and trajectory lifespans is also available as addenda to the dataset.

HARPNUM	NOAA AR#	cof	$\mu_{mindist}$	Added	Removed	Updated
6	11065	0	5.21			No
17	11071	0	0.84			No
45	11073	0	5.78		✗	Yes
182	11107	0	1.61			No
354	11151	0	0.72			No
366	11155	0	0.24			No
595	11212	0	5.10			No
619	11221	0	2.46			No
700	11253	0	5.51		✗	Yes
705	11248	0	7.50			No
705	11253	0	7.21			No
714	11256	0	4.06		✗	Yes
734	11253	0	11.06		✗	Yes
877	11293	62.71	0.17	✓		Yes
940	11326	49.34	5.54			No
973	11313	47.79	3.35			No
1041	11343	8.26	2.99			No
1124	11373	0	3.75		✗	Yes
1126	11362	4.86	7.66			No
1133	11365	0	9.18		✗	Yes
1165	11367	0	7.29		✗	Yes
1275	11392	4.42	6.92			No
1295	11394	18.15	2.62	✓		Yes
1493	11441	0	0.63			No
1493	11437	0	3.30		✗	Yes
1535	11442	85.71	0.07	✓		Yes
1578	11464	0	3.14		✗	Yes
1633	11468	96.61	0.01	✓		Yes
1662	11487	0	2.92		✗	Yes
1697	11489	0	12.73		✗	Yes
1724	11502	0	5.78		✗	Yes
1738	11492	59.85	1.66			No
1744	11504	53.77	3.12			No
1844	11522	N/A	N/A		✗	Yes
1845	11522	97.87	0.01	✓		Yes
1907	11545	0	5.68		✗	Yes
1998	11557	0	20.91		✗	Yes

2155	11594	29.41	0.53	✓		Yes
2262	11632	9.42	2.61			No
2344	11651	35.47	1.70			No
2360	11655	77.59	1.93	✓		Yes
2375	11642	0	7.23		✗	Yes
2403	11652	0	6.01		✗	Yes
2432	11666	7.96	1.70	✓		Yes
2439	11667	100	0.00	✓		Yes
2442	11665	0	4.19		✗	Yes
2469	11674	11.07	6.11	✓		Yes
2492	11679	35.4	2.04	✓		Yes
2511	11688	98.78	0.01	✓		Yes
2520	11693	65.91	1.98			No
2546	11701	100	0.00	✓		Yes
2546	11694	77.93	0.24	✓		Yes
2597	11708	77.21	2.21	✓		Yes
2666	11717	0	3.91		✗	Yes
2790	11759	0	3.60			No
2790	11761	0	4.82		✗	Yes
2878	11780	0	7.83		✗	Yes
2948	11789	0	8.26		✗	Yes
3115	11826	0	2.83		✗	Yes
3190	11844	100	0.00	✓		Yes
3212	11845	0	4.59		✗	Yes
3252	11866	0	7.81		✗	Yes
3263	11861	0	6.34		✗	Yes
3520	11935	0	5.28		✗	Yes
3520	11939	0	4.80		✗	Yes
3557	11938	0	4.64		✗	Yes
3602	11938	0	0.88		✗	Yes
3631	11958	2.08	4.08			No
3647	11965	21.61	2.50			No
3686	11975	0	17.99		✗	Yes
3744	11979	100	0.00	✓		Yes
3879	12023	5.46	7.43			No
4000	12036	0.08	14.03			No
4252	12094	0	3.13		✗	Yes
4296	12108	0	5.14		✗	Yes
4339	12112	0	0.28			No
4346	12103	54.74	1.77	✓		Yes
4390	12127	1.14	9.78			No
4424	12140	100	0.00	✓		Yes
4432	12136	33.57	1.34	✓		Yes
4438	12137	100	0.00	✓		Yes
4440	12135	100	0.00	✓		Yes
4447	12144	98.38	0.01	✓		Yes

4448	12139	96.47	0.03	✓		Yes
4450	12138	100	0.00	✓		Yes
4454	12143	100	0.00	✓		Yes
4455	12141	100	0.00	✓		Yes
4455	12142	15.44	2.98	✓		Yes
4460	12145	51.54	0.92	✓		Yes
4466	12146	100	0.00	✓		Yes
4466	12148	89.73	0.10	✓		Yes
4469	12147	88.78	0.22	✓		Yes
4477	12149	100	0.00	✓		Yes
4478	12151	100	0.00	✓		Yes
4478	12150	98.23	0.09	✓		Yes
4502	12152	98.81	0.01	✓		Yes
4505	12153	99.43	0.00	✓		Yes
4523	12161	100	0.00	✓		Yes
4523	12154	94.79	0.21	✓		Yes
4530	12157	100	0.00	✓		Yes
4530	12155	80.67	1.08	✓		Yes
4532	12156	79.79	0.18	✓		Yes
4536	12158	100	0.00	✓		Yes
4539	12163	31.46	2.00			No
4539	12159	70.79	1.16	✓		Yes
4540	12162	91.3	0.04	✓		Yes
4541	12160	100	0.00	✓		Yes
4543	12163	98.96	0.00	✓		Yes
4549	12164	98.95	0.00	✓		Yes
4552	12165	98.13	0.05	✓		Yes
4556	12166	89.19	1.46	✓		Yes
4556	12167	88.91	0.97	✓		Yes
4559	12161	13.19	7.68			No
4574	12170	97.63	0.01	✓		Yes
4574	12169	91.61	0.31	✓		Yes
4576	12168	87.39	0.16	✓		Yes
4579	12174	100	0.00	✓		Yes
4580	12171	97.59	0.16	✓		Yes
4580	12173	97.46	0.04	✓		Yes
4580	12172	96.2	0.03	✓		Yes
4591	12175	100	0.00	✓		Yes
4603	12176	93.12	0.03	✓		Yes
4616	12181	0	4.04		✗	Yes
4619	12180	21.89	3.93	✓		Yes
4645	12176	10.87	7.49			No
4661	12184	0	14.63		✗	Yes
4781	12211	28.57	1.88			No
4862	12224	0	3.39		✗	Yes
4920	12241	9.36	5.35			No

5296	12295	0	0.55			No
5321	12300	0	0.44			No
5342	12309	0	5.82		✗	Yes
5387	12312	0	17.63		✗	Yes
5434	12317	0	8.02		✗	Yes
5559	12350	1.2	5.46			No
5596	12352	0	6.51		✗	Yes
5658	12365	25.75	2.92			No
5708	12372	100	0.00	✓		Yes
5738	12383	0	3.78		✗	Yes
5750	12378	0	6.37		✗	Yes
5820	12391	8.87	2.97			No
6063	12447	0	4.36		✗	Yes
6148	12461	100	0.00	✓		Yes
6155	12467	0	2.04		✗	Yes
6178	12474	0	1.25		✗	Yes
6324	12493	0.36	7.97			No
6361	12503	0	4.71		✗	Yes
6523	12540	0	4.65		✗	Yes
6684	12568	0	1.56			No
6688	12572	0	3.99		✗	Yes
6777	12593	0	1.00		✗	Yes
6893	12622	100	0.00	✓		Yes
6901	12623	100	0.00	✓		Yes
7078	12667	100	0.00	✓		Yes
7123	12675	0	6.63		✗	Yes

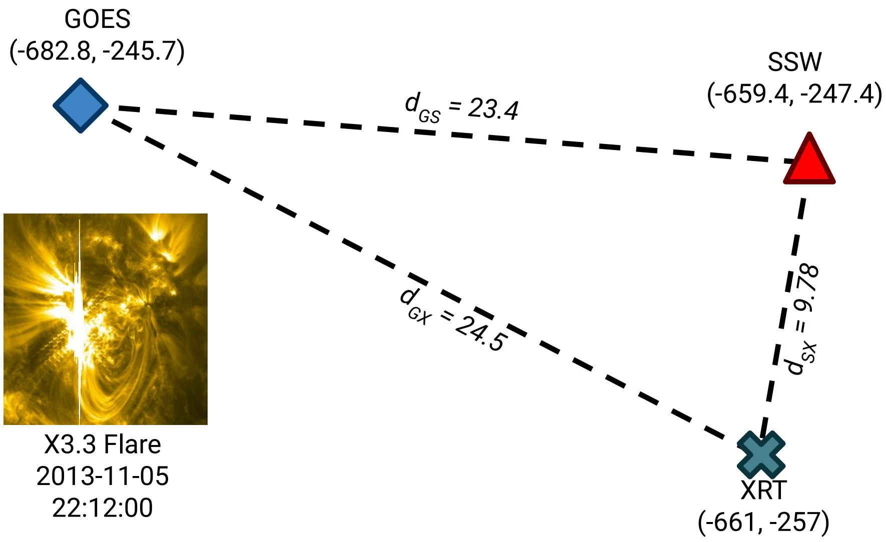

Figure S.3: Reported coordinates of the X3.3 flare occurred on 2013-11-05 at 22:12:00 from GOES, SSW Latest Events and Hinode-XRT. The Euclidean distances between these coordinates are recorded for distance-based verification. Coordinates are reported in Helioprojective Coordinate System. All locations and distances are in arcsec.

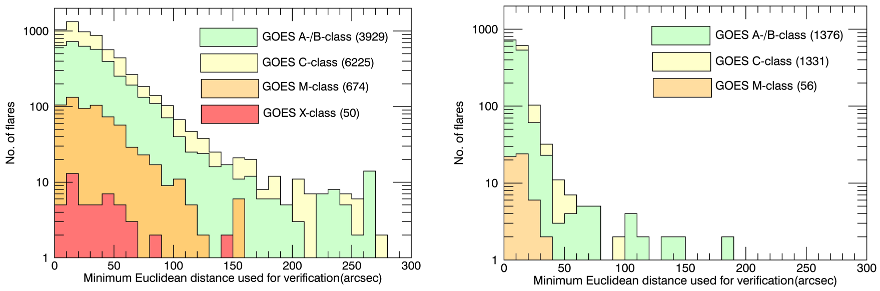

(a) (b)

Figure S.4: The distributions of the minimum distances found between GOES and SSW or XRT-reported locations for primary (a) and secondary (b) verified flares.

Date	Time	Primary	Secondary	Remarks
2017-12-12	16:30	15	14	
2017-08-24	23:20	15	13	
2017-08-17	20:00	13	14	
2016-06-09	17:30	15	13	
2016-05-16	17:00	14	15	
2016-05-12	17:30	14	13	
2016-05-03	13:00	13	14	
2015-06-09	16:25	15	13	
2015-05-21	18:00	14	13	
2015-01-26	16:01	15	13	
2012-11-19	16:31	15	None	
2012-10-23	16:00	14	15	
2011-09-01	00:00	15	14	GOES-14 data is missing from October 2011 to September 2012
2010-10-28	00:00	15	None	
2010-09-01	00:00	14	15	
2009-12-01	00:00	14	None	GOES-10 decommissioned

Table S.3: Dates and times of primary and secondary NOAA GOES satellite coverage for XRS data. Note that this table has been reproduced only for the period between 2010-05-01 and 2018-09-01, which matches the duration of our dataset. *Data courtesy:* Dr. Janet Machol.

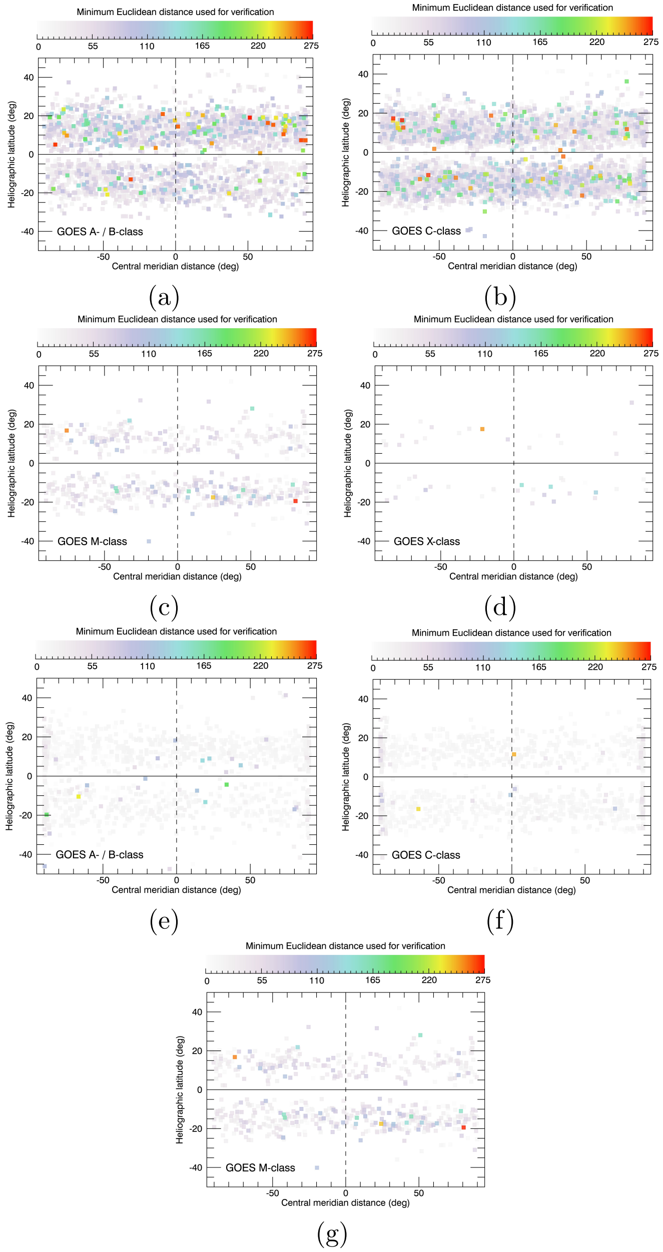

Figure S.5: The heatmaps of the minimum distances used between GOES and SSW or XRT-reported locations for primary and secondary verified flares. (a) Primary-verified A- and B-class flares. (a) Primary-verified A- and B-class flares. (b) Primary-verified C-class flares. (c) Primary-verified M-class flares. (d) Primary-verified X-class flares. (e) Secondary-verified A- and B-class flares. (f) Secondary-verified C-class flares. (g) Secondary-verified M-class flares. There are no secondary-verified X-class flares in our flare list.

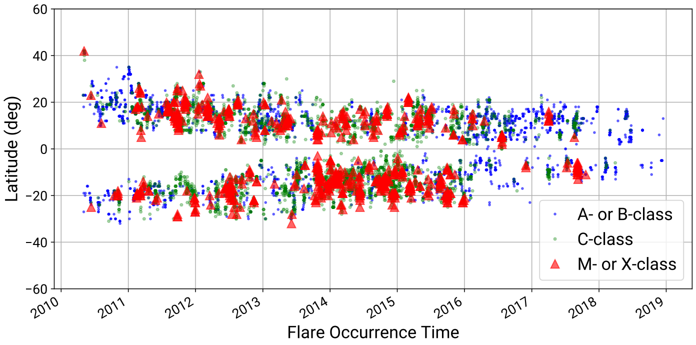

(a)

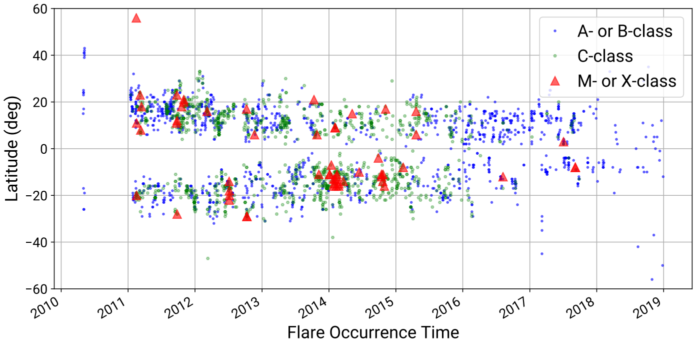

(b)

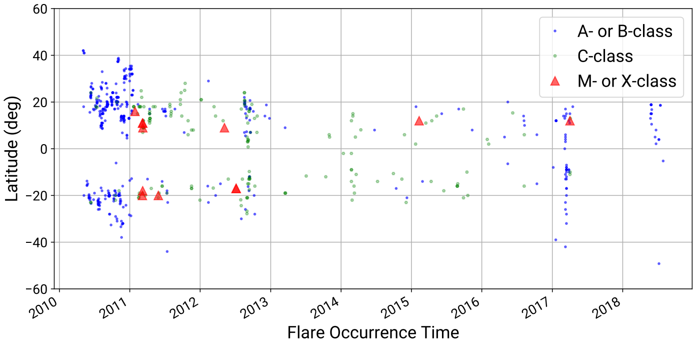

(b)

Figure S.6: The latitudes of the primary-verified (a), secondary-verified (b), and non-verified (c) flares over time.

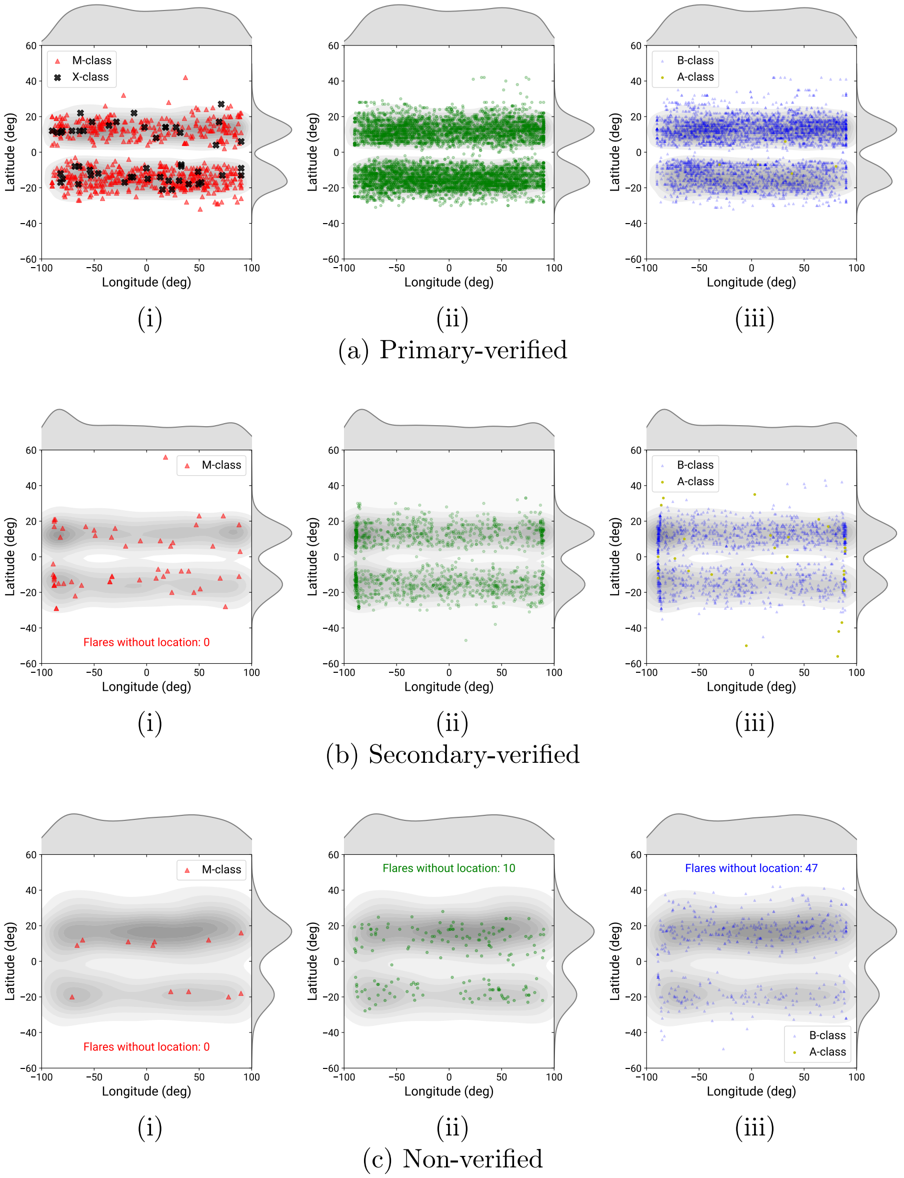

Figure S.7: The spatial distributions of the primary-verified (a)(i-ii-iii), secondary-verified (b)(i-ii-iii), and non-verified (c)(i-ii-iii) flares over time. For each verification category, M-/X-class flares, C-class flares and A-/B-class flares are shown in plots (i), (ii), and (iii), respectively. The number of flares with no valid coordinates are also annotated in each plot.

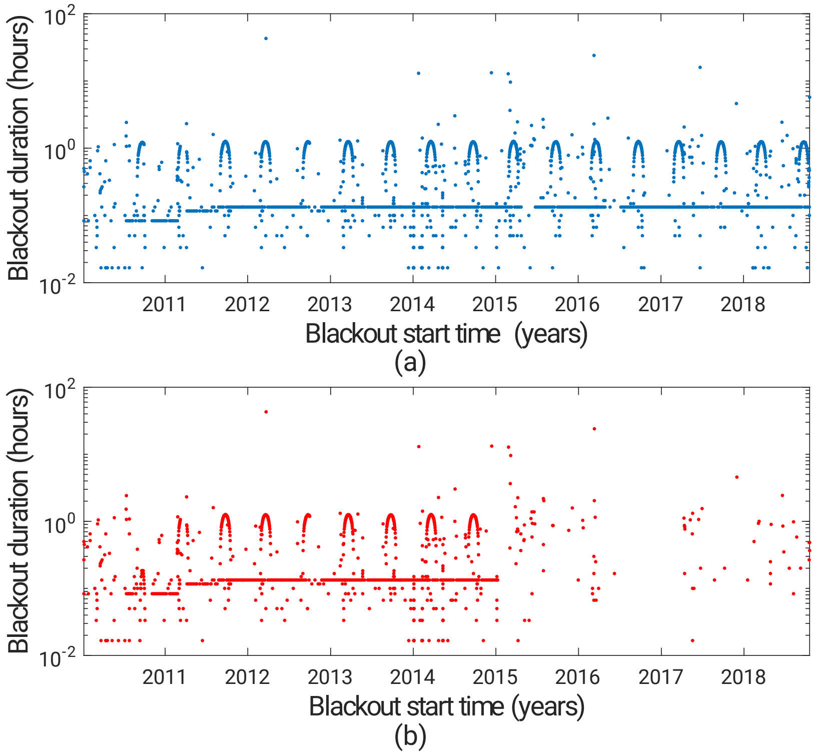

Figure S.8: (a): Blackout durations in GOES XRS data from primary satellites show a biannual periodicity. Total downtime for the period of our dataset was 1.43%. (b): After filling the gaps in primary GOES XRS data with available data from secondary satellites, the total downtime was reduced to 0.80%.
